# Distinguishing fungal and bacterial keratitis on clinical signs

**Published:** 2015

**Authors:** Astrid Leck, Matthew Burton

**Affiliations:** Research fellow: International Centre for Eye Health, London School of Hygiene and Tropical Medicine, London, UK.; Reader: International Centre for Eye Health, London School of Hygiene and Tropical Medicine, London, UK.

**Figure:**
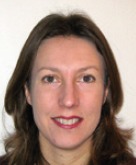
Astrid Leck

**Figure:**
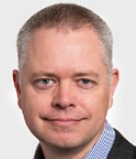
Matthew Burton

In many settings, laboratory support for the diagnosis of the type of microbial keratitis is not available. Experienced ophthalmologists have long maintained that it is sometimes possible to distinguish fungal from bacterial microbial keratitis on the basis of clinical signs. Formal data to support this view are limited, and it is important to establish the validity of such claims to understand whether signs can reliably guide clinical decisions. In addition, antifungal treatment is often in limited supply and prohibitively expensive. Therefore, it is not feasible or desirable to prescribe empirical antifungal therapy to every patient who presents with microbial keratitis in tropical regions, where fungal infections are more frequent. Here we review research to determine whether it is possible to reliably distinguish bacterial and fungal infection clinical features alone.

**‘It is not feasible or desirable to prescribe empirical antifungal therapy to every patient who presents with microbial keratitis in tropical regions, where fungal infections are more frequent.’**

In a large series from India and Ghana, cases of microbial keratitis were systematically examined for specific features.^1^ These included: serrated infiltrate margins, raised slough, dry texture, satellite lesions, hypopyon, anterior chamber fibrin, and colour. Serrated infiltrate margins and raised slough (surface profile) were independently associated with fungal keratitis, and the anterior chamber fibrin was independently associated with bacterial keratitis.^1^ Some of these features are illustrated in Figure 1. By combining information about all three features in an algorithm (Figure 2), it is possible to obtain a probability score for the likelihood that the microbial keratitis case is due to a fungus.

**Figure 1.**
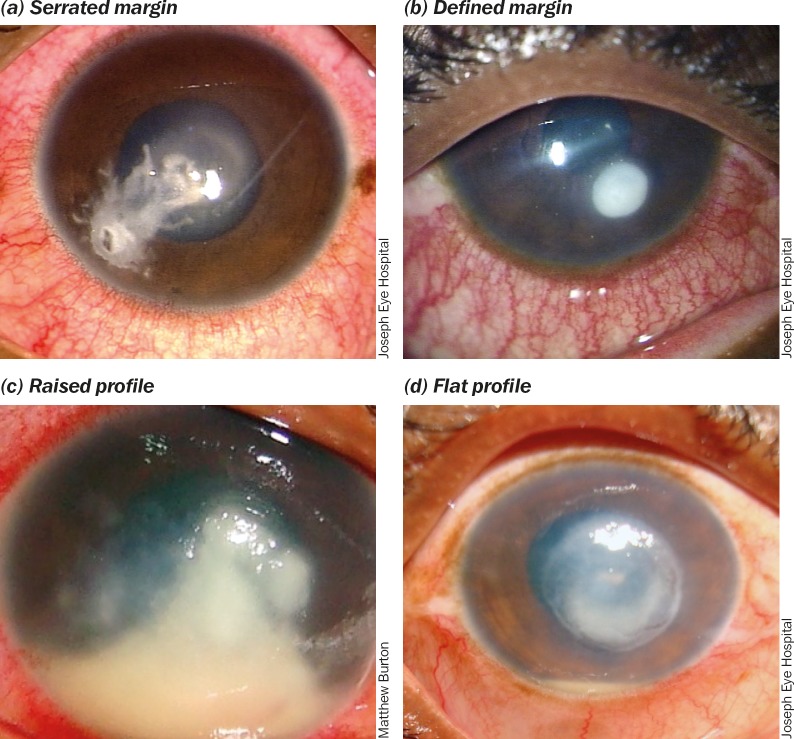
Examples key clinical features

**Challenge:** Use the algorithm (Figure 2) to estimate the probability that the microbial keratitis case in Figure 3 is due to a fungal infection. The algorithm is primarily for use as a guide in settings where clinicians do not have any laboratory facilities and treatment decisions have to be made based on clinical judgement alone. Where diagnostic microbiology is available it is strongly recommended that it is used. As discussed in the article on laboratory diagnosis in this issue, microscopy alone can provide a diagnosis if an infection is fungal; the presence of fungal hyphae in corneal tissue is a definitive diagnosis.

**Figure 2.**
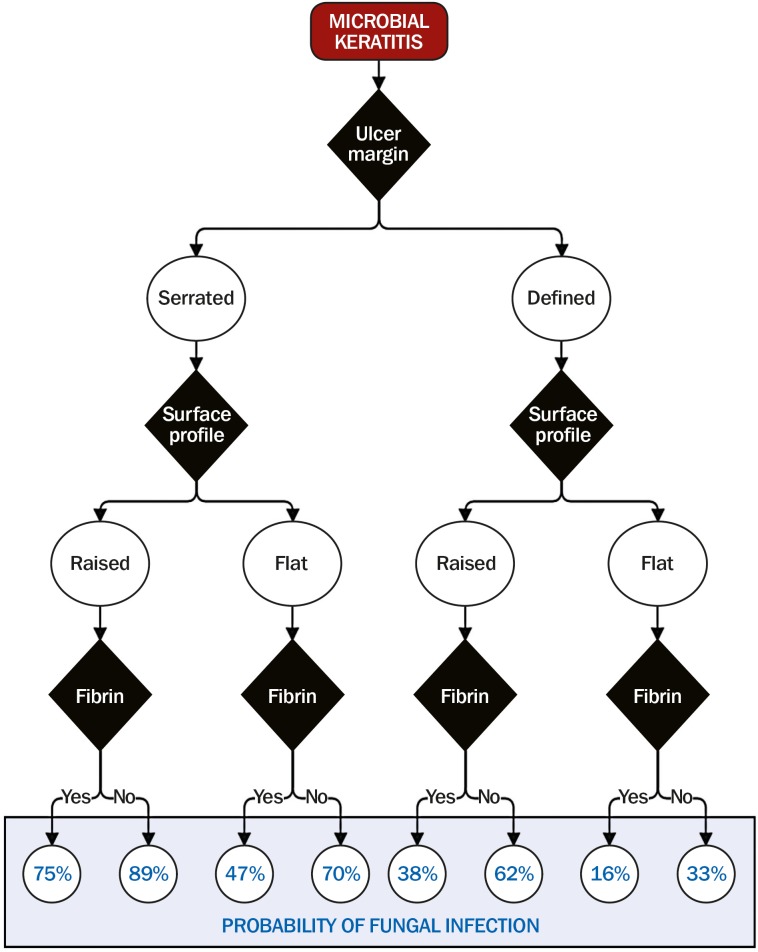
Algorithm for determining the probability of fungal keratitis. The black diamonds are decision points about three clinical features: ulcer/ infiltrate margin, surface profile, and anterior chamber fibrin. These probabilities are based on data presented in Thomas et al.^1^

**Figure 3.**
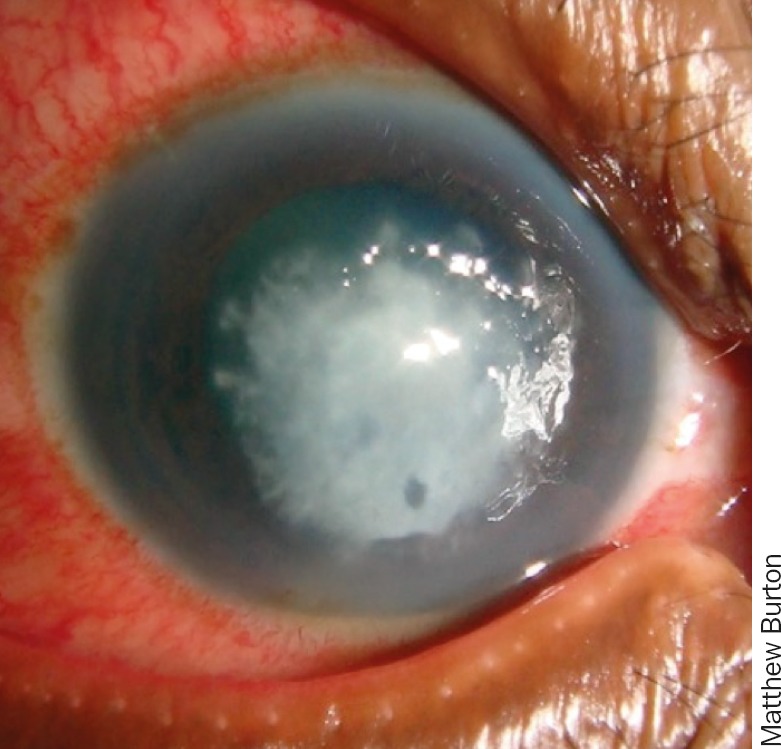
Use the algorithm (Figure 2) to estimate the probability that the keratitis is due to a fungal infection

## ANSWER

89% probability this is due to a fungal infection: serrated margin, raised profile and no anterior chamber fibrin.
